# Supplementary material for: Complexome profiling of the Chlamydomonas psb28 mutant reveals TEF5 as an early PSII assembly factor
Source: Plant Cell. 2025 Mar 17;37(6):koaf055. doi: 10.1093/plcell/koaf055 (PMC12164590; doi:10.1093/plcell/koaf055)
Supplement: koaf055_Supplementary_Data [file koaf055_supplementary_data.zip › psb28_interactive.zip!/plots/intensity/qualityPlots/peptides/PPX1_Cre09.g396300.t1.2.html]

### Cre09.g396300.t1.2

Trivial name: PPX1  
SeqCoverage [median]: 41.20  
Mass: 59.80 kDa  
  
 

| Sequence | MissedCleavage | Mass [Da] | wtIntensity | muIntensity | Score |
| --- | --- | --- | --- | --- | --- |
| AIPQFNLGHLEQLDK | 0 | 1721.91 | 1633.72 | 16493.97 | 57.80 |
| AIPQFNLGHLEQLDKAR | 1 | 1949.05 | 556.43 | 0.00 | 37.09 |
| ALDAAGLQGVHLGGNYVSGVALGK | 0 | 2266.21 | 583.22 | 975.11 | 54.22 |
| APEGHMLLLNYIGGTTNR | 0 | 1955.99 | 0.00 | 11215.42 | 84.14 |
| ASDGSVPGFGQLHPR | 0 | 1523.75 | 12277.01 | 5805.31 | 95.65 |
| AVALTAPSYVVADLVK | 0 | 1615.92 | 9467.61 | 0.00 | 120.57 |
| EADGRYGLVYDTPEGR | 1 | 1796.83 | 2882.48 | 3262.01 | 134.25 |
| GQTVGSFR | 0 | 850.43 | 11954.80 | 4699.26 | 134.81 |
| GQTVGSFRK | 1 | 978.52 | 3632.90 | 703.70 | 88.94 |
| IQNFLVTEAR | 0 | 1189.65 | 32906.17 | 14541.93 | 185.81 |
| KASDGSVPGFGQLHPR | 1 | 1651.84 | 7509.76 | 6000.38 | 81.31 |
| LRPVPSGLDAFTFDLMSIPGK | 1 | 2260.19 | 1798.40 | 0.00 | 88.07 |
| MLPDAIER | 0 | 943.48 | 8170.60 | 0.00 | 96.25 |
| NGGSLVGGAIK | 0 | 971.54 | 2140.69 | 1592.12 | 138.08 |
| NLGDEVFFR | 0 | 1095.53 | 11653.41 | 0.00 | 101.64 |
| NMVIKPDAPKPR | 2 | 1364.76 | 20205.84 | 10791.11 | 90.73 |
| QSNPAPPR | 0 | 865.44 | 249.88 | 0.00 | 71.34 |
| QSNPAPPRDPR | 1 | 1233.62 | 25295.48 | 9114.52 | 116.55 |
| RNLGDEVFFR | 1 | 1251.64 | 0.00 | 777.67 | 41.54 |
| VVEHGYESAANLAK | 0 | 1486.74 | 63072.20 | 26302.75 | 183.79 |
| YGLVYDTPEGR | 0 | 1268.60 | 23648.90 | 14403.52 | 161.27 |
|  |  |  | 239639.5 | 126678.8 |  |
